# Caffeine administration does not alter salivary α-amylase activity in young male daily caffeine consumers

**DOI:** 10.1186/1756-0500-7-30

**Published:** 2014-01-13

**Authors:** Laura Cousino Klein, Courtney A Whetzel, Jeanette M Bennett, Frank E Ritter, Urs M Nater, Michael Schoelles

**Affiliations:** 1 Department of Biobehavioral Health & Penn State Institute of the Neurosciences, The Pennsylvania State University, 219 Biobehavioral Health Building, University Park, PA 16802, USA; 2 Penn State Institute of the Neurosciences, Pennsylvania State University, University Park, PA, USA; 3 College of Information Sciences and Technology, Pennsylvania State University, University Park, PA, USA; 4 Department of Psychology, University of Marburg, Marburg, Germany; 5 Cognitive Science Department, Rensselaer Polytechnic Institute, Troy, NY, USA

**Keywords:** Salivary alpha-amylase, Caffeine, Cortisol, Blood pressure, Heart rate

## Abstract

**Background:**

To follow up on a recent report from our lab [Hum Psychopharmacol 25:359–367, 2010.] we examined the effects of caffeine on salivary α-amylase (sAA) activity in response to an engaging, non-stressful task in healthy young males (age 18–30 yrs) who consumed caffeine on a daily basis. Using a placebo-controlled, double-blind, between-subjects design, 45 men received either placebo, 200 mg or 400 mg of caffeine (Vivarin®). Participants then rested for 20 minutes, and performed a 20-minute computerized air traffic controller-like task that was cognitively engaging but not stressful. Saliva samples (assayed for sAA and cortisol), blood pressure, and heart rate were taken before (baseline) and 15 minutes after the computerized task.

**Results:**

Systolic and diastolic blood pressure and sAA activity increased across the laboratory session (F’s > 9.20, p’s < 0.05); salivary cortisol levels decreased (F = 16.17, p < 0.05). There were no main effects for caffeine administration on sAA, salivary cortisol, or cardiovascular measures, and caffeine did not interact with the task to alter these measures.

**Conclusions:**

Laboratory administered caffeine does not alter sAA activity, even when sAA activity is stimulated by participating in a cognitively engaging task. These data demonstrate that caffeine administration does not affect sAA activity, at least in healthy young men who regularly consume caffeine. Results support recent findings that basal caffeine levels in habitual caffeine users are not associated with basal sAA activity and that daily caffeine intake and diurnal sAA activity are not related.

## Background

This report is a follow up experiment to our recently published data on the effects of stress and caffeine on salivary alpha amylase (sAA) in healthy young men [1]. The health effects of caffeine and/or coffee consumption continue to be debated (e.g., [2,3]). Caffeine is a central nervous system stimulant that releases catecholamines and glucocorticoids and elevates blood pressure (see [4] for review; [5]). We recently reported that caffeine and stress together stimulate the release of salivary alpha amylase (sAA) [1], a protein involved in oral mucosal immunity [6] and carbohydrate digestion [7]. It has been suggested that sAA also is a potential biomarker of sympathetic nervous system (SNS) activity (see [8-10] for reviews). For example, we recently reported that salivary caffeine levels following caffeine and stress administration were positively correlated with sAA activity in a controlled laboratory setting [1]. These results extend correlational field study finding that self-reported daily caffeine intake, along with stress exposure, is associated with increased sAA levels [11,12].

Caffeine and stress may stimulate sAA activity via SNS activation (for review, see [13]). Caffeine bioavailability may be altered in unanticipated ways as a result of stress exposure on the pharmacokinetic profile of caffeine and its metabolites, though this area needs further investigation (e.g., [14]). Therefore, it is possible that stress exposure in the presence of caffeine administration may interact in important physiological ways that alter sAA activity. Stress in the absence of caffeine results in increased sAA activity (for review see [9]), but the effects of caffeine on sAA activity in the absence of stress are not widely known.

Recent data suggest that caffeine alone may not alter sAA activity [1]. We reported that basal caffeine levels in habitual caffeine users do not appear to be associated with basal sAA activity [1]. Further, daily caffeine intake does not appear to affect diurnal sAA patterns [13]. However, the direct effects of caffeine on sAA activity remain unknown. Therefore, we conducted a controlled laboratory experiment to examine the effects of caffeine administration on sAA activity in healthy young men who regularly consumed caffeine (i.e., at least 50 mg of caffeine per day). Participants were asked to complete a computerized task that was engaging but not stressful to allow for the examination of caffeine’s influence on sAA without activating a classic stress response as indicated by hypothalamic-pituitary-adrenal axis activity (i.e., salivary cortisol elevations [15]). We hypothesized that the task, but not caffeine, would increase sAA activity.

## Methods

### Participants

Forty-five healthy men (18–30 years old; M = 21.24 ± 0.39 years), were recruited to participate in a study examining caffeine and task performance through flyers posted in the local community and on the Penn State campus. Sex differences in caffeine pharmacokinetics can alter caffeine metabolism and absorption in unanticipated ways [16], therefore, we only included men in this initial study. Interested individuals were screened by a trained research assistant in a telephone interview that reviewed health history. Eligibility criteria were identical to those in a previous study reported by our laboratory [1]. Specifically, participants were daily caffeine users who consumed at least 50 mg of caffeine per day (e.g., 8 oz cup of coffee, 12 oz can caffeinated soda). Respondents were excluded for significant health problems and the use of medications or drugs that could affect interpretation of neuroendocrine or cardiovascular data, could alter caffeine metabolism, or potentially could harm the participant if caffeine were administered, including history of: smoking or nicotine use, chronic medical conditions, medication use for any chronic physical and/or mental health conditions, and medications that could alter salivary biomarker data (e.g., oral or parenteral corticosteroids within prior 3 months, over-the-counter stimulants, flu/cold medications, caffeine- or ephedrine-containing supplements, cimetidine, quinolones, verapamil). Further, individuals with obesity (greater than 140% of ideal body weight) were excluded as determined by body mass index (BMI; weight/height^2^) > 30 [17] confirmed during the lab visit. The Center for Epidemiological Studies Depression Scale (CES-D) [18] was administered to screen (and exclude) for symptoms of depression.

Mean BMI did not differ across experimental groups (see Table 1). Seventy-six percent of the participants were Caucasian (N = 34), 11% were African American (N = 5), 9% were Asian (N = 4), and 4% were self-described as “other” (N = 2). Ethnicity was equally represented across the three experimental groups [χ^2^(6, 45) = 0.28, n.s.]. All participants were high school graduates; 91% of participants had completed some college, and 9% had earned higher than a college degree.

**Table 1:** Age and body mass indices (BMI) of men in each caffeine treatment group (means ± SEM)

	**Caffeine treatment Ggroups**		
	**Placebo (N = 15)**	**200 mg (N = 15)**	**400 mg (N = 15)**
Age (years)	22.00 ± 0.60	20.67 ± 0.58	21.07 ± 0.80
Body mass index (kg/m^2^)	23.43 ± 0.70	23.64 ± 0.80	23.11 ± 0.57

### Experimental protocol

#### Overview

The experimental protocol is identical to a previously published experiment with the exception of the air traffic controller task described below [1]. Eligible participants refrained from taking daily vitamins on the day of their session, ate a low-fat lunch by 1100 hrs, and avoided caffeine consumption 4 hours prior to their lab session. Participants arrived at the Penn State University General Clinical Research Center (GCRC) on the day of their lab session. All sessions started at 1300 hrs. Following informed consent by a trained research assistant (CAW or JMB), a certified nurse practitioner confirmed health status and study eligibility. Participants then were asked to complete a demographic survey and comprehensive measure of daily caffeine use. Next, a standard blood pressure cuff (Dinamap Compact Blood Pressure Monitor, Critikon, Tampa, FL) was placed on the non-dominant arm to collect systolic blood pressure (SBP), diastolic blood pressure (DBP), and heart rate (HR). Blood pressure values from this automated oscillometric blood pressure monitor are highly, positively correlated with intra-arterial and ambulatory blood pressure measurements [19,20]. After cuff placement, a sample blood pressure reading was taken to ensure that blood pressure levels fell within an acceptable range (i.e., SBP <140 mmHg, DBP <90 mmHg, HR <100 beats per minute). All participants met these criteria.

#### Baseline

Participants were asked to sit quietly for 10 minutes while 5 baseline blood pressure readings were taken automatically at 2-minute intervals. Participants next were asked to give a saliva sample by rolling a cotton swab across their tongue (without chewing on the swab) for 2 minutes and then placing it into a saliva collection tube (Salivette; Sarstedt, Inc., Newton, NC). Saliva samples immediately were placed on ice until transferred to a minus 80 degree low-temperature freezer for later assessment of sAA and cortisol. Participants then were asked to complete three computerized tasks that measured working memory, reaction time, and visual attention (results not reported here), during which time blood pressure and HR were recorded every 2 minutes. Together, these 3 computerized tasks took 3–5 minutes to complete.

#### Caffeine administration

Following the computer tasks, participants were asked to swallow two gelatin capsules with a glass of water. Details about the capsules are reported in Klein et al. [1]. Each capsule contained either methylcellulose (placebo; Spectrum Chemicals, Gardena, CA) or a 200 mg Vivarin® (GlaxoSmithKline, Philadelphia, PA) pill. Using a randomized double-blind procedure, participants in the placebo group (N = 15) received two methylcellulose capsules, participants in the 200 mg caffeine group (N = 15) received 1 methylcellulose and 1 caffeine capsule, and participants in the 400 mg caffeine group (N = 15) received 2 caffeine capsules. This caffeine administration paradigm was selected based on previously published studies (e.g., [1,21,22]) and to parallel caffeine consumption outside the lab where individuals consume caffeine in the form of beverages (e.g., sodas, coffee) and food (e.g., chocolate).

Based on our prior research [1], participants waited 20 minutes following capsule administration to allow for adequate caffeine absorption. This rest period ensured that participants completed the computerized task when plasma caffeine levels were on the ascending limb of the absorption curve [23,24]. Blood pressure and HR readings were taken every 2 minutes. This caffeine administration method and the timing of saliva collection results in a dose-dependent increase in caffeine and caffeine metabolite levels [1].

#### Air traffic controller task

The air traffic controller task (i.e., Argus; [25]) is an interactive, engaging task requiring cognitive, perceptual, and motor processing. Moving targets appear on the computer screen and the subject must classify as many targets as possible in 2, 10-minute-sessions. The task took 20 minutes to complete; blood pressure and HR were recorded every minute during this time period.

#### Recovery

Participants next repeated the 3 computerized tasks that they completed during baseline (i.e., working memory, reaction time, signal detection), rest for a 15-minute recovery period to allow us to capture potential hormone changes following the air traffic controller task [1,26], and then provide a second saliva sample. Blood pressure and HR were recorded every 2 minutes throughout this recovery period. At the end of the session, participants were paid $50 and a GCRC nurse informed them of their caffeine condition. All procedures were reviewed and approved by The Pennsylvania State University Institutional Review Board.

### Physiological measures

#### Salivary cortisol

On the day of assay, samples were allowed to thaw to room temperature and then were centrifuged at 1500 × g for 15 minutes to remove mucins and other particulate matter that could interfere with the assay. Salivary cortisol levels were assayed at the GCRC using commercially-available enzyme immunoassay kits (DSL, Webster, TX). The sample test volume was 25 ul. The assay had a lower limit of sensitivity of 0.011 ug/dl, with an average inter- and intra-assay covariance (%) of less than 8% and 5%, respectively. The assay sensitivity (0.011 ug/dl) is based on the minimum cortisol concentration required to produce a three standard deviation from assay A_0_. Samples from each participant were tested in duplicate in a single assay batch. Values used in data analyses are the averages of duplicate tests.

#### Salivary α-amylase (sAA)

Salivary α-amylase was measured at Salimetrics, LLC (State College, PA) using a kinetic reaction assay [27]. The sample test volume was 10 ul. The assay had an average inter- and intra-assay covariance (%) of less than 6% and 7.5%, respectively. Saliva samples from each participant were tested in singlet in a single assay batch per assay guidelines.

#### Blood pressure and heart rate

Systolic blood pressure, DBP, and HR readings were averaged across each experimental time period to derive mean baseline (5 readings), challenge (16 readings), and recovery (6 readings) measures for each participant [1].

### Statistical analyses

Square root transformations were applied to the sAA data because they were not normally distributed [1,8]; this transformation resulted in a normal distribution of the data. Similarly, log-transformation of the cortisol data resulted in a normal distribution [1,28]. Thus, all sAA and cortisol statistical analyses are based on transformed values; raw sAA and cortisol values are reported unless otherwise noted.

Separate repeated-measures analysis of variance (RM-ANOVA), with Caffeine Treatment (3 levels) as the independent measure and Time as the within-subject variable, were conducted to examine group differences in sAA and cortisol levels during the baseline and recovery phases of the experiment. A multivariate RM-ANOVA that included SBP, DBP, and HR as the dependent measures was used to examine group differences across baseline, task performance, and recovery. When appropriate, statistical interactions were examined using separate one-way ANOVAs, Tukey’s honestly significant difference (HSD), and Bonferroni *post-hoc* analyses. All significance tests were two-tailed and evaluated at α = 0.05.

## Results

### Blood pressure and heart rate

Systolic and diastolic blood pressure increased across the laboratory session such that SBP and DBP levels did not return to baseline among all participants [F(2,84) > 9.20, p’s < 0.05] (see Table 2). In contrast, HR levels dropped across the session [F(2,84) < 9.80, p < 0.05] for all participants. There were no main effects for caffeine administration on SBP, DBP, or HR levels nor did caffeine interact with time to alter these cardiovascular measures.

**Table 2:** Laboratory-administered caffeine dosage (mg/kg) and baseline, task performance, and recovery systolic (SBP) and diastolic (DBP) blood pressure (mmHg) and heart rate (beats per minute; BPM) levels among men in each caffeine treatment group (estimated marginal means ± SEM)

	**Caffeine treatment groups**		
	**Placebo (N = 15)**	**200 mg (N = 15)**	**400 mg (N = 15)**
*Caffeine dosage* (*mg/kg*)	0.00 ± 0.00	2.64 ± 0.09^1^	5.51 ± 0.28^1^
*Systolic blood pressure* (*mmHg*)			
Baseline	112.76 ± 2.06	116.76 ± 2.06	116.45 ± 2.06
Task performance	113.54 ± 2.44	116.26 ± 2.44	121.02 ± 2.44
Recovery	114.93 ± 2.20^2,3^	120.35 ± 2.20^2,3^	122.20 ± 2.20^2,3^
*Diastolic blood pressure* (*mmHg*)			
Baseline	65.16 ± 1.67	65.33 ± 1.67	65.43 ± 1.67
Task performance	65.55 ± 1.91	67.98 ± 1.91	68.99 ± 1.91
Recovery	70.73 ± 1.74^2,4^	70.73 ± 1.74^2,4^	70.93 ± 1.74^2,4^
*Heart rate* (*BPM*)			
Baseline	64.84 ± 2.29	65.49 ± 2.29	67.25 ± 2.29
Task performance	66.52 ± 2.24	65.19 ± 2.24	68.74 ± 2.24
Recovery	64.13 ± 2.00^2,5^	61.73 ± 2.00^2,5^	63.56 ± 2.00^2,5^

^1^Caffeine treatment effect (200 mg < 400 mg), p < 0.0001.

^2^Time effect, p < 0.05.

^3^Baseline = Task Performance < Recovery, p < 0.05.

^4^Baseline < Task Performance < Recovery, p < 0.05.

^5^Baseline = Task Performance > Recovery, p < 0.05.

### Salivary cortisol

**Figure 1:**
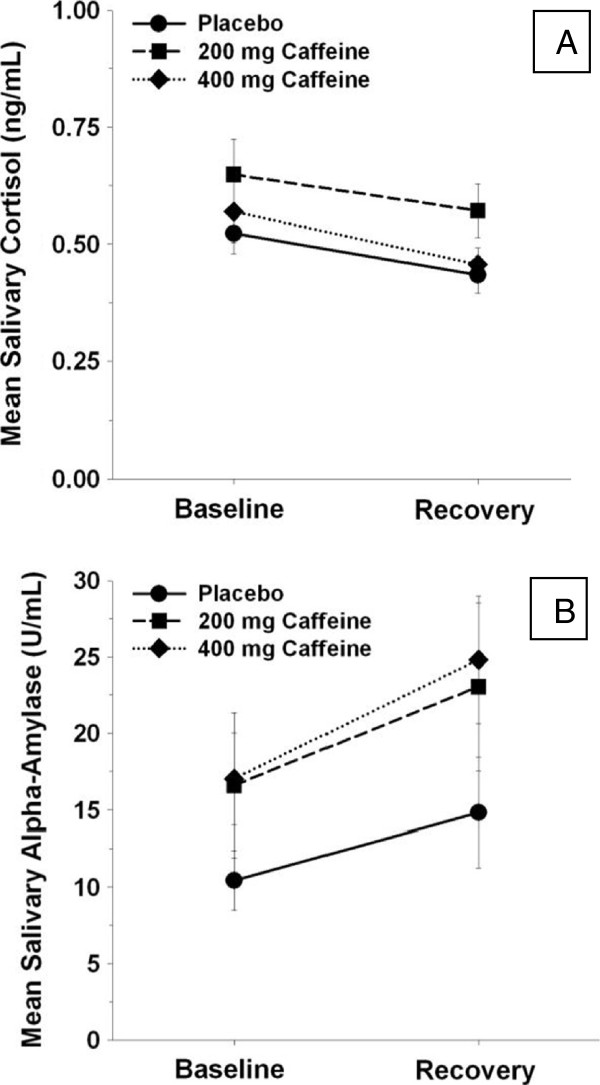
**Salivary cortisol (ng/mL) and salivary alpha-amylase (U/mL) among men administered no caffeine, 200 mg or 400 mg of caffeine at baseline (before caffeine administration) and recovery (15 min following cessation of air traffic controller) (unadjusted means + SEM). (A)** Mean salivary cortisol levels **(B)** mean salivary alpha-amylase levels.

Baseline salivary cortisol levels did not differ among the caffeine groups and cortisol levels decreased across the laboratory session across all participants [F(1,42) = 5.77, p < 0.05]. Caffeine administration did not alter salivary cortisol levels and there was no time X caffeine group interaction (Figure 1).

### Salivary α-amylase (sAA)

Baseline sAA activity also did not differ among the caffeine groups. Overall, the increased sAA activity across all participants [F(1,42) = 16.17, p < 0.05]. However, similar to cortisol, caffeine did not alter sAA activity and there was no statistically significant caffeine X time interaction.

## Discussion

We examined the effects of caffeine administration on salivary α-amylase (sAA) activity in response to an engaging, non-stressful task in healthy young men who consumed caffeine on a daily basis (i.e., at least 50 mg of caffeine). Participants were asked to engage in a computerized task that did not elicit a hypothalamic-pituitary-adrenal axis stress response (i.e., salivary cortisol). We found that laboratory administered caffeine does not alter sAA activity, even when sAA activity is stimulated by participating in a cognitive computer task. These data support recent findings that basal caffeine levels in habitual caffeine users are not associated with basal sAA activity [1] and that daily caffeine intake and diurnal sAA activity are not related [13]. Our results are the first to demonstrate that caffeine administration does not affect sAA activity, at least in healthy young men who regularly consume caffeine.

The lack of a cortisol or cardiovascular response to caffeine administration is consistent with earlier reports that caffeine’s endocrine and/or cardiovascular effects may be most apparent in vulnerable populations such as individuals at risk for hypertension in the presence of a stressor (e.g., [29,30]). In addition, cardiovascular responses can attenuate to repeated caffeine exposures such as daily consumption, suggesting that nonsignificant blood pressure and heart rate responses to the caffeine manipulation may be the result of habituation [31,32].

This study included a limited number of indices of the sympathetic nervous system (sAA, blood pressure, heart rate) across two time points. As activity and reactivity of these measures is also influenced by the parasympathetic branch of the autonomic nervous system (ANS) one might consider adding purely sympathetic measures (such as electrodermal activity) in future research. Such additional laboratory-based experiments are needed to delineate the mechanisms through which caffeine may or may not alter ANS activity. These studies should have a more inclusive ANS panel of biomarkers, such as plasma catecholamines and heart rate variability, and include caffeine administration both with and without stress across individuals. Further, men and women should be included in these studies, along with varying caffeine dosages, and multiple ANS assessments across the day.

## Conclusions

Laboratory administered caffeine does not alter sAA activity, even when sAA activity is stimulated by participating in a cognitively engaging task. These data demonstrate that caffeine administration does not affect sAA activity, at least in healthy young men who regularly consume caffeine. Results support recent findings that basal caffeine levels in habitual caffeine users are not associated with basal sAA activity and that daily caffeine intake and diurnal sAA activity are not related.

## Competing interests

None of the authors have competing financial or personal interests to disclose.

## Authors’ contributions

LCK and FER obtained funding and designed the experiment; CAW and JMB played a significant role in data acquisition; LCK analyzed the data and drafted first version of manuscript; LCK, CAW, JMB and FER played significant roles in data analyses. MS developed the Argus task. All authors made substantive and significant contributions to data interpretation and manuscript content. All authors made critical revisions to the manuscript and read and approved the final manuscript.
